# Hypomagnesaemia and other electrolytes imbalances in open and closed pediatrics cardiac surgery

**DOI:** 10.12669/pjms.35.2.367

**Published:** 2019

**Authors:** Mohsen Shahidi, Houman Bakhshandeh, Khaled Rahmani, Abdorrahim Afkhamzadeh

**Affiliations:** 1 *Mohsen Shahidi, Pediatric Cardiologist, Department of Pediatric Cardiology, Rajaiee Heart Center, Tehran, Iran*; 2 *Houman Bakhshandeh, Department of Research, Rajaiee Heart Center, Valiasr Ave, Tehran, Iran*; 3 *Khaled Rahmani, Assistant Professor of Epidemiology, Social Determinants of Health Research Center, Research Institute for Health Development, Kurdistan University of Medical Sciences, Sanandaj, Iran*; 4 *Abdorrahim Afkhamzadeh, Associate Professor of Community Medicine, Social Determinants of Health Research Center, Research Institute for Health Development, Kurdistan University of Medical Sciences, Sanandaj, Iran*

**Keywords:** Cardiac, Operation, Hypomagnesaemia

## Abstract

**Objective and Background:**

During the past decade, many researchers have indicated that open cardiac surgery, using cardiopulmonary bypass, could be an essential factor to induce post-operative electrolyte imbalances which may be followed by life threatening complications such as arrhythmia. Nevertheless, by this time there may be a few researches about comparing of hypomagnesaemia and other electrolyte imbalances between open, on pump, and closed, off pump, heart operation.

**Methods:**

In this cohort study conducted at Rajaie Heart Center in Tehran from December 2014 to August 2015, we evaluated hypomagnesaemia, hypocalcemia, hypokalemia and hyponatremia in 205 children aged under 15 years who underwent open (101 children) and closed (104 children) cardiac surgery. Repeated measures ANOVA, paired t test and Chi-square/Fisher exact test were used for analysis the data in SPSS version 21.

**Results:**

According to our study the frequency of electrolyte imbalances including hypomagnesaemia after pediatric heart surgery is relatively high (28.7% hypomagnesaemia at the second day) with more occurrence in closed cardiac operations. There was no significant relationship between hypomagnesaemia and pump time duration (P>0.05). On the other hand, this research indicated that there is significant relationship between post-operative hypomagnesaemia and some other variables including cyanotic heart disease (P=0.01) and concurrent electrolyte imbalance such as hypocalcaemia and hypokalemia (P<0.05).

**Conclusion:**

Early evaluation and correction of hypomagnesaemia should be considered after both closed and open heart operation.

## INTRODUCTION

Electrolyte disturbances are prevalent in hospitalized patients. Based on exist evidence, electrolyte disturbances especially hypomagnesaemia is one of the most important causes of post-operative cardiac arrhythmia.^1^,^2^ This arrhythmia may be resistant to the anti-arrhythmia drugs.^3^

Magnesium is an intracellular cation. It is an essential element which catalyses more than 300 enzymatic reactions. Serum magnesium determination represents only 1% of total body’s magnesium concentration. Modern instruments will soon be available to determine physiologically active intracellular ionized magnesium.^4^

Hypomagnesaemia, as one of the most important electrolyte imbalances, in patients who undergo cardiac surgery can be due to food insufficient intake or as a result of increased magnesium excretion due to diuretics, cardiac glycosides and use albumin or citrated blood products. Moreover, the level of magnesium during and after the use of a cardiopulmonary pump in adults and children decreases.^5^

Electrolyte imbalances can emerge after operation and has been associated with higher incidence of both postoperative arrhythmias and low cardiac index.^2^,^6^ In infants with congenital cardiac lesions hypomagnesaemia was reported above 40%.^7^ To our knowledge, there is no strength evidence about difference between two type of cardiac surgery, open/on-pump or closed/off-pump, regarding electrolyte imbalances particularly hypomagnesaemia. The aim of this study was to assess the electrolyte imbalances in children undergoing heart surgery and differentiate between open and closed procedures in electrolyte imbalances.

## METHODS

This was a prospective cohort study that was approved by research committee of Rajaie Heart Center which is licensed via Iranian health minister. Pediatric cardiology department of this hospital is the main referral center in Iran for complex congenital heart disease. No informed consent was obtained in our study.

We aimed to compare serum electrolyte level with especial impression to hypomagnesaemia after both open/on pump, and closed/off pump, heart operation. In addition, simultaneous assessment of other electrolytes including calcium, sodium and potassium were considered for their association with hypomagnesaemia and also with defined predictors.

We enrolled 205 patients, who needed to heart surgery, including 100 girls and 105 boys under 15 years old from December 2014 to August 2015 in Rajaie Heart Center in Tehran. Our exclusion criteria were patients with chronic renal problems such as renal failure and renal tubular acidosis, gastro-intestinal disorders like chronic diarrhea or malabsorption, hypoalbuminemia and congenital or nutritional rickets. All patients who were undergoing open (101 children) or closed (104 children) heart surgery during December 2014 to August 2015 followed for three days after surgery. Baseline data of study subjects including demographic characteristics, type of surgery, and electrolytes measurements were collected by trained personnel through a checklist from pediatric heart wards before operation which was followed in the intensive care unit after heart surgery during 1^th^ hour, 2^nd^ and 3^rd^ day of operation.

During open heart surgery, prime solution consisted of; 6-10 ml/kg of colloid solution plus lactated ringer to a total volume of oxygenator capacity (e.g. 1L). Synthetic colloid solution contains; succinylated gelatin 0.902 gr/L, calcium 0.2.88meq/L, sodium 80.7meq/L, chloride 80.7meq/L and ringer lactate contains; sodium 130 mmol/L, cloride 109 mmol/L, potassium 4 mmol/L, calcium 1.5 mmol/L, lactate 28 mmol/L. Packed red blood cell was added according to the patient’s hematocrit. No additional magnesium was administered during cardiopulmonary bypass.

For cardiac arrest, diluted crystalloid cardioplegia, adding ringer solution to total volume of one litter was administered 15-20 ml/kg. Non diluted crystalloid cardioplegia contains; magnesium chloride 3.25gr/20ml and potassium chloride 1.193gr/20ml. Therefore, the estimated administered magnesium chloride during open heart surgery was about 50-60mg/kg.

### Laboratory measurements

Laboratory tests including serum magnesium and other electrolytes were performed in four stages started before surgery, early after surgery followed by second and third day after operation. Total serum level was measured for magnesium, calcium, potassium and sodium.

Serum magnesium level of 1.6 to 2.6 mg/dl was considered normal. Hypomagnesaemia was considered if serum magnesium level was equal or under 1.6 mg/dl. Hypomagnesaemia corrected immediately after detection with intravenous magnesium sulfate 30 mg/kg of over 30 minutes.

Likewise, other electrolyte imbalances especially calcium were treated according to the laboratory data. Normal total calcium level was considered between 8.6 to 10.5 mg/dl. Hyponatremia and hypokalemia were defined as the serum levels of lower than 135 and 3.5 mmol/L, respectively. Hypocalcaemia was corrected with administration of one ml/kg of a 10% solution of calcium gluconate every six to eight hours. Symptomatic hyponatremia was corrected by administration of 4-6 ml/kg of 3% sodium chloride solution with considering serum sodium level and in cases with hypovolemic states by compensating both water and sodium deficits. Mild hypokalemia (3-3.5mmol/L) was compensated through increasing of maintenance intravenous potassium from 20 up to 80 mmol/L and lower serum levels were usually corrected by administration of 0.5 mmol/kg of potassium chloride during one to two hours.

Serum albumin level was evaluated for all cases before and after surgery every day until discharge from pediatric intensive care unit. It was within normal range in relatively all study patients before operation; however, postoperative hypoalbuminemia was immediately corrected by administration of 20% albumin solution. All study patients had normal renal function which evaluated by blood urea nitrogen and serum creatinine.

### Statistical Analysis

Mean ± standard deviation and frequency (%) were used to describe continuous and categorical data, respectively. Variations between serum magnesium (and other electrolytes) in different times and between two study groups (open and closed heart surgery) was investigated and control for other predictors was performed by repeated measures analysis of variance (ANOVA) models. P value < 0.05 was considered as statistically significant. SPSS version 21 was used for statistical analysis.

## RESULTS

A total of 205 patients, 105 (51.2%) boys and 100 (48.8%), were included in the study. Mean and standard deviation of age was 4.22±4.55 years. Of total 205 patients 122 (59.5%) were under three years old. Mean serum potassium was relatively declined to 4.2, 4.3 and 4.2 mmol/L for the 1th hour, 2d and 3rd day of operation, respectively, in comparison to preoperative period (4.6mmol/L) without remarkable relationship with other variables (Table-I).

**Table-I:** Changes in level of magnesium, calcium, potassium and sodium of serum over the time.

	Measuring time				

	before operation	1^th^ hour after operation	2^ed^day after operation	3^rd^day after operation	P value
Mg	2.30±0.57	2.25±0.80	1.82±0.34	1.83±0.33	P<0.001
Cal	9.67±0.92	7.92±1.03	8.77±0.83	8.86±0.69	P<0.001
K	4.57±0.66	4.19±0.38	4.34±0.48	4.18±0.28	P<0.001
Na	139.12±9.54	138.35±10.01	136.47±3.53	135.53±2.72	P<0.001

Hypomagnesaemia was present just in five patients (2.3%) before operation but laboratory data in the first hour and also second and third day after surgery displayed 25(12.4%), 62(30.4%) and 46(27.7%) of patients with hypomagnesaemia, respectively. Mean magnesium serum level before operation was 2.3 mg/dl which decreased to 2.2, 1.81 and 1.83 at the 1th, 2d and 3d day after operation, respectively (P<0.001). Changes in serum level of magnesium, calcium, potassium and sodium over the time along with results of statistical significance of these changes are summarized in Table-II.

**Table-II:** Changes in level of Magnesium, Calcium, Potassium and Sodium of serum over the time.

Measuring time						

		Before operation	1th hour after operation	2nd day after operation	3rd day after operation	P value
Open heart surgery (n=101)	Mg	2.32±0.59	2.58±0.88	1.84±0.36	1.81±0.41	P<0.001
	Cal	9.85±0.89	7.65±1.10	8.78±0.78	8.88±0.69	P<0.001
	K	4.38±0.45	4.25±0.34	4.32±0.48	4.13±0.27	P<0.001
	Na	140.49±3.53	140.03±13.61	137.28±3.37	135.64±2.69	P<0.001
Closed heart surgery (n=104)	Mg	2.30±0.55	1.92±0.56	1.79±0.32	1.84±0.22	P<0.001
	Cal	9.49±.92	8.19±0.89	8.75±0.89	8.84±0.68	P<0.001
	K	4.76±0.77	4.14±0.41	4.36±0.48	4.24±0.28	P<0.001
	Na	137.80±12.83	136.72±3.61	135.68±3.53	135.43±2.74	P=0.01

As shown in Table-II, magnesium changes over time are significant (P <0.001). Linearity trend of changes was observed and statistically significant (P<0.001). There was no statistical association between magnesium changes over time and sex (P=0.3) and methods of surgery, open or close (P=0.7). Results of magnesium changes in two groups, open and closed surgery, separately shown in Table-III. It should be noted that sphericity assumption was checked during conducting of repeated measurement and sphericity was assumed.

**Table-III:** Association between type of surgery and frequency of electrolyte disturbance.

			Open heart surgery, n (%)	Closed heart surgery, n (%)	P value
Hypomagnesaemia
	Before operation	Yes	1 (20.0)	4 (80.0)	0.4*
		No	100 (50.0)	100 (50.0)	
	1th hour after operation	Yes	12 (27.9)	31 (72.1)	0.002
		No	89 (54.9)	73 (45.1)	
	2nd day of operation	Yes	28 (45.2)	34 (54.8)	0.4
		No	73 (51.0)	70 (49.0)	
	3rd day of operation	Yes	32 (69.6)	14 (34.0)	0.002
		No	69 (43.4)	90 (56.6)	
Hypocalcemia	
	Before operation	Yes	5 (25.0)	15 (75.0)	0.03
		No	96 (51.9)	89 (48.1)	
	1th hour of operation	Yes	87 (55.4)	70 (44.6)	0.002
		No	14 (29.2)	34 (70.8)	
	2nd day of operation	Yes	42 (45.7)	50 (54.3)	0.3
		No	59 (52.2)	54 (47.8)	
	3rd day of operation	Yes	33 (54.1)	28 (45.9)	0.4
		No	68 (47.2)	76 (52.8)	
Hypokalemia	
	Before operation	Yes	1 (25.0)	3 (75.0)	0.6*
		No	100 (49.8)	101 (50.2)	
	1th hour after operation	Yes	0 (0)	10 (100.0)	0.002*
		No	101 (51.8)	94 (48.2)	
	2nd day of operation	Yes	1 (16.7)	5 (83.3)	0.2*
		No	100 (50.3)	99 (49.7)	
	3rd day of operation	Yes	1 (100.0)	0 (0)	0.5*
		No	100 (49.0)	104 (51.0)	
Hypernatremia	
	Before operation	Yes	9 (32.1)	19 (67.9)	0.06
		No	92 (52.0)	85 (48.0)	
	1th hour after operation	Yes	5 (12.8)	34 (87.2)	<0.001
		No	96 (57.8)	70 (42.2)	
	2nd day of operation	Yes	30 (35.7)	54 (64.3)	0.001
		No	71 (58.7)	50 (41.3)	
	3rd day of operation	Yes	48 (56.5)	37 (43.5)	0.08
		No	53 (44.2)	67 (55.8)	

* Fisher’s Exact Test result

**Fig.1:**
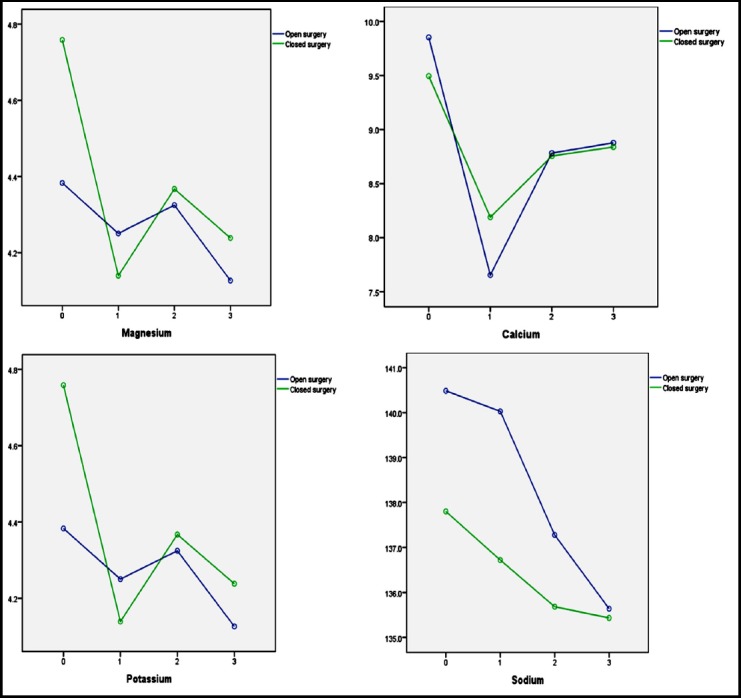
Changes in parameters in two study groups, open and closed heart surgery (0= before operation, 1= 1th hour after operation, 2= 2nd day after operation, 3rd day after operation)

Since there was significant relationship for interaction between type of surgery, open and closed, and hypomagnesaemia, hypocalcaemia, hypokalemia and hypernatremia in Repeated Measures ANOVA, we analyzed the frequency of these electrolytes decrease in subgroup of surgery. Table-II and Fig.1 show the results of subgroup analysis.

As shown in Table-III, occurrence of hypomagnesaemia and hypokalemia on first day after surgery in children undergoing closed surgery was significantly higher than open surgery (P<0.05), whereas occurrence of hypocalcemia and hyponatremia was significantly higher in open surgery group (P<0.05). Although decrease in four parameters (Mg, K, Ca and Na) during second and third days of surgery is seen but difference between two type of surgery except Mg was not statistically significant. Surprisingly frequency of hypomagnesaemia on third post-operative day was higher in children with open surgery.

Considering that the total administered magnesium during open heart surgery has been about 50-60mg/kg of magnesium chloride as the crystalloid cardioplegia. Likewise, mean pump time was not a predictor of hypomagnesaemia (P=0.4).

Frequency of cyanotic and non-cyanotic patients were 84 (41%) and 121 (53%) respectively. Comparing with non-cyanotic heart disease, there was statistical relationship between hypomagnesaemia and cyanotic group at the second day of operation with acceptable confidential interval (P=0.001).

Simultaneous evaluation of serum level of other electrolytes including calcium, sodium and potassium before and after surgery and their association with hypomagnesaemia was another part of our study. Pair sample test displayed that mean total serum calcium level declined statistically (P<0.001) from the preoperative value of 9.7mg/dl to early postoperative level of 7.9. Interestingly, hypocalcaemia was significantly more frequent in comparison with hypomagnesaemia at the first hour after operation (55.5% versus 28%), nonetheless; its rate gradually decreased during the succeeding days. Though, simultaneous association between hypomagnesaemia and hypocalcaemia was present during the third day of operation. In other words, during the third postoperative day, 20% of cases (33 patients) had both hypomagnesaemia and hypocalcaemia (P=0.007).

We compared frequency of hypocalcaemia between groups of open and closed heart surgery. In addition to high frequency of hypocalcaemia at the first hour after operation it was statistically significant among “on pump” group (P<0.001) but during the following days it was relatively equal among both groups. here was also borderline statistical relationship between hypocalcaemia and pump time duration at the first hour after surgery (P=0.05).

Hypocalcaemia among cyanotic group was statistically significant on the second day in comparison to noncyanotic (P<0.001). It should be noted that serum magnesium level was corrected with single infusion of magnesium sulfate in 48% and two times in 39% and three times in 13%.

## DISCUSSION

In this prospective cohort study we analyzed the electrolyte imbalances between two heart surgery methods, open and closed. Based on the results, one of the most important electrolyte changes was hypomagnesaemia after heart surgery that has been occurred in 43 (21%) of the patients at one hour after surgery. Our results are compatible with some previous reports.^4^

The important viewpoint is increasing frequency of hypomagnesaemia during proceeding days especially the second day and occurrence of new cases of hypomagnesaemia during second and third days that all display presence of factors other than cardiopulmonary bypass.

Through comparion of two groups of open and closed heart surgery, significant association was found during twice after surgery, hence frequency of hypomagnesaemia in patients who had undergone closed surgery was higher at first hour after operation while no significant relationship was observed between two groups on second day and interestingly hypomagnesaemia was significantly higher in open surgery group. Bearing in mind that the sole perioperative recommended magnesium was through cardioplegia solution, this is important particularly in comparing with other researches.^7^,^8^ Therefore, it could be concluded that some other pre and post-operative metabolic issues in addition to cardiopulmonary bypass should be involved in postoperative hypomagnesaemia. For instance, intracellular shift of magnesium due to acidosis, blood transfusion and epinephrine, insulin, glucose release or administration and refeeding syndrome may cause hypomagnesaemia.^9^,^10^ On the other hand, intraoperative ischemia and reperfusion injury may result in release of magnesium into extracellular fluid early after surgery.^11^,^12^

Some studies have reported that acid-base abnormality could induce hypomagnesaemia.^11^,^12^ It has long been known that systemic acidosis is associated with renal magnesium wasting.^13^ Calcium and magnesium balance was maintained by concurrent intestinal absorption, renal excretion and exchange with bone. Recently, calcium and magnesium transport proteins responsible for divalent cations balance have been identified.^13^,^14^ Similar reports may make a clue to explain the association of the hypocalcemia and hypomagnesaemia.^15^

Since hypomagnesemia has been associated with other electrolyte abnormalities, the detection of hyponatremia, hypokalemia, hypophosphatemia, or hypocalcemia may indicate the possibility of coexisting hypomagnesemia, vice versa other electrolyte abnormalities are frequently encountered in patients with hyponatremia of any origin.^16^,^17^

Considering that congestive heart failure is relatively common after cardiac surgery arouses the suspicion of its possible role to cause electrolyte imbalances through activation of the renin-angiotensin-aldosterone system. The most commonly encountered are hyponatremia, hypokalemia and hypomagnesemia.^18^

One study has reported that patients with hypomagnesaemia and hypocalcemia display acid base and electrolyte abnormalities especially hypokalemia due to hyperkaliuria, hypophosphatemia and acid base disorders. Urinary depletion of potassium sometimes gives rise to magnesium and calcium excretion.^19^

In conclusion, according to the previous researches electrolyte imbalances especially hypomagnesaemia could be an important cause of some complications particularly arrhythmia after heart surgery.^19^ Although, some previous studies have emphasized on relationship between hypomagnesaemia and open heart surgery, this study, is proposing that high frequency of hypomagnesaemia should be considered after both closed and open pediatric heart surgery. Our study showed that there is a significant association between serum electrolyte level and surgery types in different follow-up times. We recommend the measuring and consideration of serum electrolytes especially magnesium during and after pediatric cardiac surgery.
